# Thoracic Ossification of the Ligamentum Flavum With Isolated Radiculopathy in a Teenage Baseball Pitcher: Possibly the Youngest Surgically Treated Case

**DOI:** 10.7759/cureus.84531

**Published:** 2025-05-21

**Authors:** Masaru Nakano, Kinshi Kato, Kenichi Otoshi, Takuya Nikaido, Kazuyuki Watanabe, Hiroshi Kobayashi, Yoshihiro Matsumoto

**Affiliations:** 1 Department of Orthopedic Surgery, Fukushima Medical University, School of Medicine, Fukushima, JPN; 2 Department of Orthopedic Surgery, Department of Sports Medicine, Fukushima Medical University, School of Medicine, Fukushima, JPN; 3 Department of Sports Medicine, Fukushima Medical University, School of Medicine, Fukushima, JPN; 4 Department of Research for Spine and Spinal Surgery, Department of Orthopedic Surgery, Fukushima Medical University, School of Medicine, Fukushima, JPN

**Keywords:** adolescent, baseball, case report, ligamentum flavum, ossification, thoracic spine

## Abstract

Ossification of the ligamentum flavum (OLF) typically occurs in individuals aged >50 years, especially in East Asian men, and commonly presents with myelopathy. Cases in adolescents, especially athletes, are exceedingly rare. We report a case of thoracic OLF in a 19-year-old right-handed collegiate baseball pitcher who presented with isolated radiculopathy. The patient experienced sharp left dorsal pain (numerical rating scale 6-8/10) during the late cocking phase of pitching, which involves thoracic extension, rotation, and slight left lateral flexion. Despite one year of conservative treatment, including physical therapy and adjustments to pitching mechanics, his symptoms persisted, ultimately leading to a performance breakdown often referred to as the "yips". He subsequently underwent surgical resection of the ossified ligament at the T8-T9 level, resulting in rapid symptom resolution and a full return to competitive sports. To our knowledge, the current case represents the youngest patient with surgically treated thoracic OLF to date. This case underscores the importance of considering thoracic OLF in the differential diagnosis of unilateral thoracic pain in young throwing athletes and illustrates the role of surgery when pain impairs athletic function.

## Introduction

Ossification of the ligamentum flavum (OLF), a pathological condition characterized by ectopic bone formation within the ligamentum flavum, primarily affects the thoracic spine in East Asian men aged ≥50 years [1-3]. The ligamentum flavum contributes to posterior spinal stability and helps preserve normal spinal biomechanics during movement [3]. Repetitive stress may induce ligamentous hypertrophy, which can lead to ossification in predisposed individuals. OLF is typically associated with thoracic myelopathy; however, isolated radiculopathy may rarely occur. Recently, case reports have highlighted a new trend of young, high-level baseball pitchers developing thoracic OLF, likely due to repetitive mechanical loading and asymmetrical spinal stress [4-9].

To our knowledge, no surgically treated cases of thoracic OLF in patients aged <20 years have been reported. This case report describes a 19-year-old collegiate pitcher with thoracic radiculopathy due to OLF, who was successfully managed with surgical decompression. It also discusses the clinical and biomechanical implications specific to adolescent athletes.

## Case presentation

A 19-year-old right-handed male collegiate baseball pitcher presented with a one-year history of progressive left dorsal pain radiating to the anterior chest region. The pain first began at 18 years of age, without any identifiable trauma, and gradually worsened. Intercostal neuralgia was initially diagnosed, for which he underwent conservative management, including rest, physical therapy, and medications. He had no history of spinal injury, spinal surgery, systemic disorders, or family history of ossification of the posterior longitudinal ligament or ligamentum flavum.

The patient began playing baseball at age seven and initially served as a right-handed catcher in youth leagues. He transitioned to pitcher at age 12 upon joining a hardball program. At 15, he entered a nationally prominent high school with a long history of success in national tournaments, where he was listed on the official bench roster as an elite pitcher. This reflected sustained exposure to high-level training and competition throughout adolescence.

**Figure 1:**
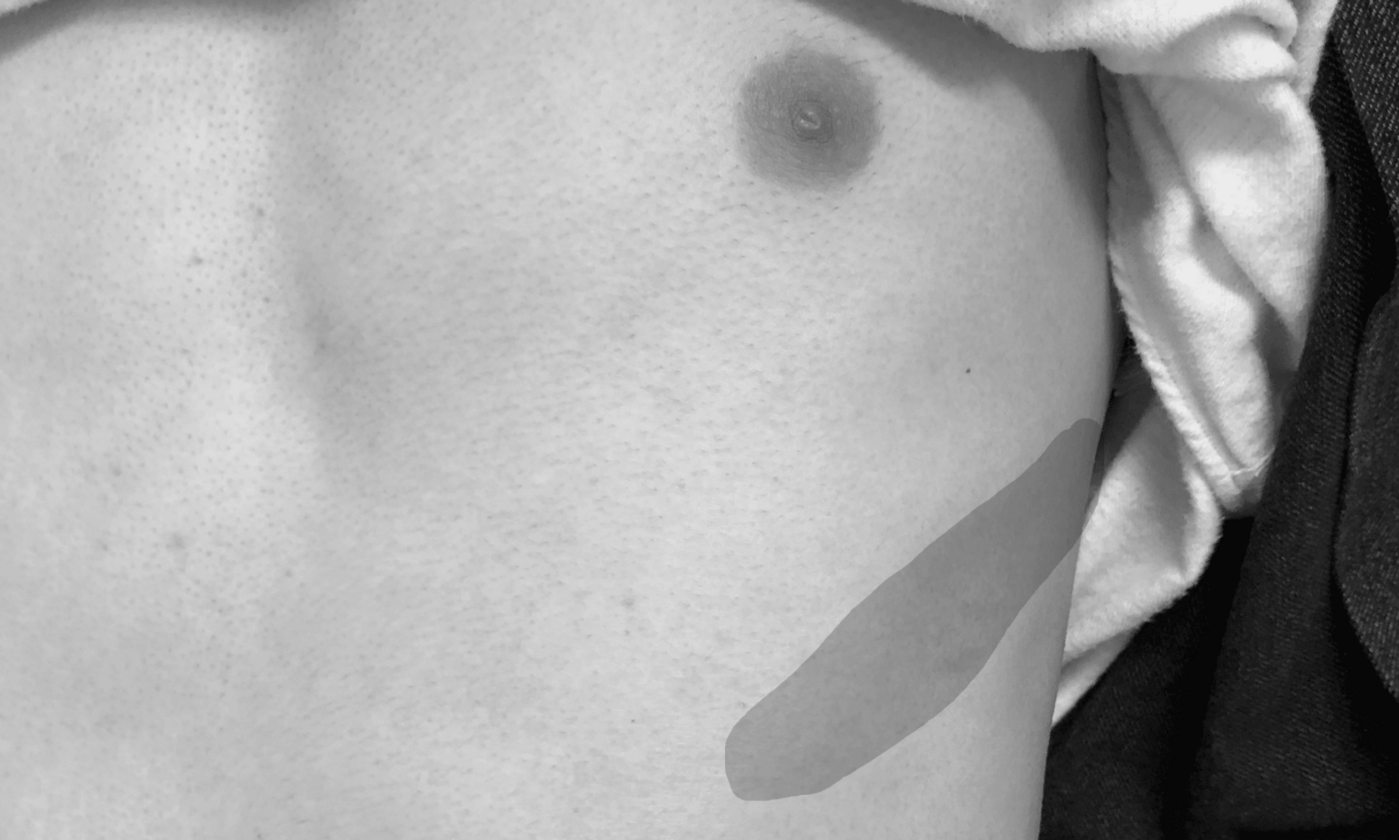
Clinical photograph showing the area of sensory disturbance over the left anterior chest wall. The shaded region (light gray) indicates the patient-defined distribution of hypoesthesia, corresponding to the T8 dermatome. This area was identified through bedside neurological examination and marked in collaboration with the patient to reflect his actual symptoms.

Neurological examination revealed hypoesthesia in temperature and pain sensation in the left T8 dermatome, consistent with T8 radiculopathy (Figure 1). Although the sharpened Romberg test results were positive, there were no motor deficits, pathological reflexes, or signs of myelopathy.

**Figure 2:**
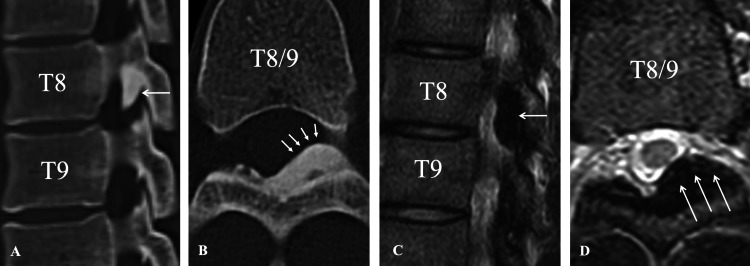
Preoperative CT and MRI images demonstrating left-sided ossification of the ligamentum flavum at T8-T9 A) Sagittal CT image showing localized hypertrophic ossification of the ligamentum flavum and foraminal narrowing at the T8-T9 level (arrow); B) Axial CT revealing localized ossification at the T8-T9 level (arrows); C) Sagittal T2-weighted MRI showing localized hypertrophic ossification of the ligamentum flavum and foraminal narrowing at the T8-T9 level (arrow); D) Axial T2-weighted MRI showing left-sided foraminal stenosis without spinal cord compression (arrows) CT - computed tomography; MRI - magnetic resonance imaging

Computed tomography (CT) and magnetic resonance imaging (MRI) of the thoracic spine were obtained to evaluate for thoracic nerve root compression, given the persistence of localized dorsal and anterior chest wall pain with dermatomal distribution. Imaging revealed left-sided hypertrophic ossification of the ligamentum flavum at the T8-T9 level, compressing the left exiting nerve root within the intervertebral foramen (Figure 2). Spinal cord compression was not observed, leading to a diagnosis of thoracic radiculopathy due to OLF.

Conservative management included rest, physical therapy focusing on shoulder and trunk mechanics, NSAID use for symptom control, and stepwise return-to-throwing attempts. Corticosteroid injections or nerve blocks were not attempted, as symptoms were consistently provoked only during high-level pitching. Over the course of a year, the patient attempted to modify his pitching mechanics to reduce thoracic extension and rotation during the late cocking phase, when his symptoms were most pronounced. Specific adjustments included lowering his arm slot (release point) and deliberately minimizing thoracic left lateral flexion, both of which were aimed at reducing mechanical stress on the symptomatic region. Despite these adjustments, the pain persisted and interfered with his throwing control, ultimately resulting in task-specific motor dysfunction ("yips"). The diagnosis of the yips was based on the patient's self-reported loss of fine motor control during pitching, observed deterioration in throwing accuracy, and persistent anxiety despite preserved physical ability. No formal psychological assessment tools were applied. Given the physical and psychological impact, surgical intervention was ultimately chosen.

Prior to surgery, the patient reported no pain at rest but experienced sharp left dorsal pain (numerical rating scale (NRS): 6-8/10) during the late cocking phase of pitching, which involves thoracic extension, rotation, and slight left lateral flexion. On bedside physical examination, thoracolumbar flexion, extension, rotation, and side-bending did not reproduce the symptoms. The straight leg raise was 90° on the right and 95° on the left. Hip internal and external rotations were symmetric bilaterally (30° and 60°, respectively). The heel-buttock distance was 0 cm on both sides.

**Figure 3:**
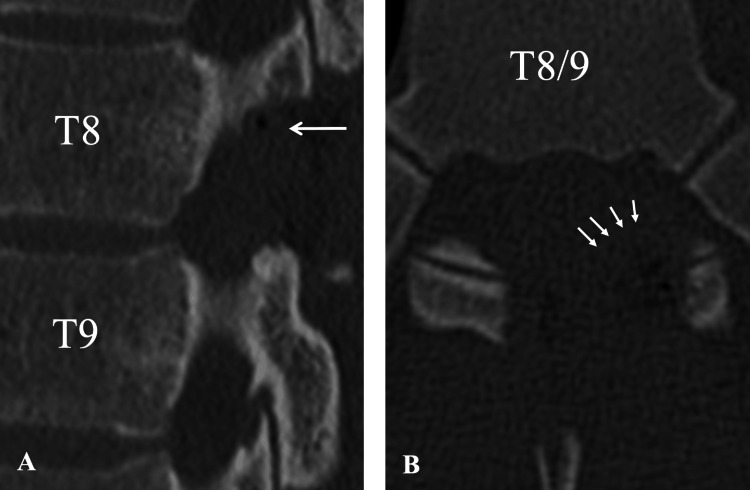
Postoperative CT images following left-sided partial laminectomy and resection of the ossified ligamentum flavum at T8-T9 A) Sagittal CT view demonstrating restoration of the intervertebral foramen with no residual ossification (arrow); B) Axial CT image confirming complete resection of the ossified ligamentum flavum and decompression of the foraminal stenosis at T8-T9 (arrows) CT - computed tomography

During the procedure, only the left paraspinal muscle was detached to expose the T7-T8 spinous processes and T8-T9 laminae. A left-sided T8-T9 partial laminectomy and foraminotomy with ossified ligament resection were performed (Figure 3). The O-arm and StealthStation S7 (Medtronic, Minneapolis, MN) were employed as CT navigation systems to confirm the location of the OLF during decompression. Intraoperatively, the affected nerve root was significantly compressed, but no adhesion to the dura was noted. The surgery was completed with no complications.

Postoperatively, the dorsal pain resolved entirely by postoperative day one (NRS 0/10), and the anterior chest paresthesia resolved by day five. Postoperative rehabilitation included trunk stabilization (drawing-in, abdominal bracing) and lower-extremity exercises, such as bodyweight squats, heel raises, and front lunges, which were initiated on postoperative day five. Exercises were performed twice daily in three to five sets of 10-15 repetitions. Conditioning progressed to include light jogging and net throwing at two weeks, followed by catch-ball training at three to four weeks. Fielding drills such as grounder work and random tosses were introduced from week four onward. Concurrently, progressive aerobic conditioning (medium-distance runs) was implemented.

At week 10, the athlete advanced to controlled bullpen pitching and a full conditioning program, including shuttle runs and trunk rotation under load. Full-intensity mound pitching was permitted after 14 weeks, contingent upon the absence of pain and normalization of balance and proprioception. The patient achieved his personal best pitch velocity of 147 km/h at 24 weeks.

At one-year follow-up, the patient remained symptom-free, and there was no evidence of OLF recurrence or adjacent-level pathology on imaging. He ultimately retired from competitive baseball at 16 months postoperatively. This decision was not due to persistent symptoms or physical limitations but rather reflected the structural realities of elite baseball in Japan. Only a small proportion of collegiate pitchers advance into professional or semi-professional leagues. Although residual performance anxiety related to the yips may have influenced his confidence, his surgical outcome was considered successful from both neurological and functional perspectives.

## Discussion

OLF is a relatively rare cause of spinal canal or foraminal stenosis that primarily affects men aged >50 years, particularly within East Asian populations [1,2]. It predominantly involves the lower thoracic spine and typically presents with myelopathy, characterized by progressive gait disturbances, spasticity, and bladder dysfunction [3]. Symptomatic OLF is exceedingly rare in teenagers; previously, the youngest reported patient was a 20-year-old female (non-athlete) with extensive multilevel thoracic OLF that developed following mild back trauma [10]. To our knowledge, the current case represents the youngest patient with thoracic OLF who has been surgically treated to date.

Recent reports have increasingly identified young, high-level athletes, especially baseball pitchers, as a distinct at-risk group for early-onset thoracic OLF [4-9]. During the late cocking and acceleration phases of pitching, the thoracic spine undergoes combined extension, axial rotation, and lateral flexion, movements that place a considerable mechanical load on the posterior elements, particularly at the thoracolumbar junction [4]. Recent biomechanical studies have shown that torsional torque transferred from the pelvis to the upper torso significantly contributes to trunk rotation during pitching. In particular, Kimura et al. reported that the mechanical energy transferred through thoracic torsional torque averaged 0.72 ± 0.19 J/kg/m, underscoring the magnitude of rotational forces acting on the thoracic spine [11]. These repetitive, high-velocity movements may cause cumulative microtrauma to the ligamentum flavum, predisposing it to hypertrophy and ossification. The thoracic spine, particularly at the T8-T12 levels, serves as a critical axis for trunk rotation and stabilization during throwing. Our patient, a right-handed pitcher with left-sided thoracic OLF, exemplifies this mechanical stress model, similar to previous cases in pitchers in their 20s [4-7]. This contrasts with OLF in the general elderly population, where multifactorial causes, including genetics, metabolic disorders, and systemic ossification tendencies (e.g., diffuse idiopathic skeletal hyperostosis) have been implicated [1-3]. The unilateral manifestation of OLF has been noted in several reports, and it is hypothesized that asymmetric torsional stress and microtrauma applied during the late cocking and acceleration phases of pitching may create repetitive strain on the contralateral ligamentum flavum [4]. This biomechanical asymmetry, combined with localized spinal kinematics, may promote unilateral hypertrophy and eventual ossification, even in the absence of a systemic predisposition. Although a genetic predisposition cannot be entirely excluded, given the higher prevalence of OLF in East Asian populations, our patient had no family history of ossification disorders or evidence of systemic ossification on imaging.

In this case, the clinical presentation was isolated thoracic radiculopathy without signs of myelopathy, which is a relatively uncommon manifestation. Young athletes with OLF may exhibit a range of neurological symptoms, from radiculopathy to overt myelopathy. In several baseball pitchers, the initial complaint was unilateral leg pain (buttock, thigh, or calf) accompanied by numbness, resembling lumbar radiculopathy rather than the classic midline signs of thoracic myelopathy [4,6,8]. This can result in diagnostic delays, as the lumbar spine is often the initial focus of investigation. In severe cases, bilateral leg weakness, numbness, and bladder and bowel dysfunction have been observed, indicating significant spinal cord compression [5]. Conversely, some athletes display very subtle cord involvement; for instance, two professional pitchers (both left-handed and in their 20s) experienced only thoracic axial pain with mild sensory changes and no clear motor deficits [7]. This suggests that OLF can occasionally lead to predominantly foraminal or nerve root compression (radiculopathy) with minimal direct cord impingement, depending on the size and location of the ossification. Other potential causes of thoracic radiculopathy in athletes, such as thoracic disc herniation [12], costovertebral joint dysfunction, or rib stress injury, should be considered in the differential diagnosis, especially when imaging findings are equivocal.

Although OLF-associated radiculopathy can sometimes be managed conservatively [7], early surgical decompression should be considered for high-performance athletes when pain significantly disrupts biomechanics and sport-specific performance. In the present case, the possibility of recovery through conservative treatment was explained to the patient, and this influenced his initial decision to avoid surgery. However, after approximately one year of persistent symptoms during pitching, surgery was performed to facilitate a return to competitive sport. Timely decompression not only alleviates symptoms but may also prevent irreversible motor maladaptation and psychological deterioration associated with prolonged dysfunction. In this case, persistent pain during thoracic rotation interfered with the patient's pitching mechanics. Multiple compensatory adjustments led to a progressive loss of control, ultimately resulting in task-specific dysfunction ("yips"), which necessitated surgical intervention. While earlier surgical intervention might have reduced prolonged dysfunction and the psychological burden of delayed recovery, it is not possible to determine whether this would have prevented the development of the yips. The relationship remains speculative. In this case, retirement was likely multifactorial and not solely attributable to medical delay.

This case underscores that OLF is not solely a degenerative disease of aging; it can develop much earlier in individuals subjected to chronic localized spinal stress. Although a genetic predisposition cannot be entirely ruled out, the absence of ossification in other spinal regions and the unique mechanical loading pattern suggest a primary stress-induced etiology in this patient. As spinal imaging becomes increasingly common among young athletes presenting with back or radicular symptoms, additional cases of early-onset OLF may be identified. Increased awareness among sports medicine specialists, spine surgeons, and athletic trainers is essential for timely diagnosis, preventing permanent neurological impairments, and preserving athletic careers. Although comprehensive epidemiological data on OLF in athletes are lacking, case series indicate a trend toward unilateral, often left-sided, presentation among baseball pitchers in their 20s and 30s [4-9]. These cases suggest a pattern of sport-specific mechanical vulnerability that may warrant early screening in high-risk populations. This case also illustrates how delayed intervention in high-level athletes may have psychological consequences that impact long-term athletic performance. Future cases may benefit from a multidisciplinary approach that addresses both physical and mental aspects of recovery.

A limitation of this report is the absence of comprehensive standardized outcome instruments, such as validated functional scores or sports anxiety questionnaires. While the NRS was documented, future case studies may benefit from incorporating broader psychological and functional measures to more accurately capture the physical and mental dimensions of postoperative recovery in athletes. Additionally, recent studies have suggested possible genetic contributions to thoracic OLF, including mutations in COL6A1 [13]. Although our patient had no family history or evidence of systemic ossification, genetic testing was not performed, and a hereditary component cannot be excluded. Although repetitive torsional stress from pitching may contribute to localized ligamentous ossification, a direct causal link between pitching mechanics and the development of OLF has not been definitively established. Further studies are needed to clarify this potential relationship. Finally, as this is a single-case report, the findings are not generalizable. Selection bias may be present, and future research with larger cohorts will be necessary to validate and expand upon these observations.

## Conclusions

Thoracic OLF is typically regarded as a degenerative condition affecting older adults; however, this case demonstrates that it can also arise in younger individuals subjected to repetitive mechanical stress, such as young throwing athletes. Clinicians should include thoracic OLF in the differential diagnosis of adolescents presenting with persistent dorsal or lateral chest wall pain, particularly when symptoms mimic radiculopathy and persist despite conventional management.

Early surgical intervention should be considered when pain impairs performance, especially in high-level athletes, as timely decompression may prevent secondary motor dysfunction and psychological deterioration. Although our patient achieved full neurological and functional recovery, he ultimately retired from competition, highlighting that athletic outcomes depend on physical, psychological, and systemic factors. These cases may benefit from a multidisciplinary approach involving spine surgeons, sports medicine specialists, athletic trainers, and mental health professionals to address both biomechanical and psychological recovery.

Future research should investigate long-term outcomes in young athletes and explore preventive strategies for high-risk individuals, including screening protocols such as the Sharpened Romberg test, assessment of thoracic flexibility and throwing mechanics, and early MRI imaging for persistent dorsal or thoracic radiculopathy. A larger case series is currently underway to address these issues.
